# Serum proteome profiles in cats with chronic enteropathies

**DOI:** 10.1111/jvim.16743

**Published:** 2023-06-06

**Authors:** Jane Yu, Lara Boland, Melissa Catt, Leah Puk, Nadia Wong, Mark Krockenberger, Peter Bennett, Craig Ruaux, Valerie C. Wasinger

**Affiliations:** ^1^ Sydney School of Veterinary Science, Faculty of Science The University of Sydney Sydney New South Wales 2006 Australia; ^2^ Paddington Cat Hospital Paddington New South Wales Australia; ^3^ McIvor Road Veterinary Centre Bendigo Victoria Australia; ^4^ Bioanalytical Mass Spectrometry Facility, Mark Wainwright Analytical Centre University of New South Wales Sydney New South Wales Australia

**Keywords:** alimentary small cell lymphoma, biomarkers, chronic enteropathies, low‐grade alimentary lymphoma, proteins, proteomics

## Abstract

**Background:**

Serum protein biomarkers are used to diagnose, monitor treatment response, and to differentiate various forms of chronic enteropathies (CE) in humans. The utility of liquid biopsy proteomic approaches has not been examined in cats.

**Hypothesis/Objectives:**

To explore the serum proteome in cats to identify markers differentiating healthy cats from cats with CE.

**Animals:**

Ten cats with CE with signs of gastrointestinal disease of at least 3 weeks duration, and biopsy‐confirmed diagnoses, with or without treatment and 19 healthy cats were included.

**Methods:**

Cross‐sectional, multicenter, exploratory study with cases recruited from 3 veterinary hospitals between May 2019 and November 2020. Serum samples were analyzed and evaluated using mass spectrometry‐based proteomic techniques.

**Results:**

Twenty‐six proteins were significantly (*P* < .02, ≥5‐fold change in abundance) differentially expressed between cats with CE and controls. Thrombospondin‐1 (THBS1) was identified with >50‐fold increase in abundance in cats with CE (*P* < 0.001) compared to healthy cats.

**Conclusions and Clinical Importance:**

Damage to the gut lining released marker proteins of chronic inflammation that were detectable in serum samples of cats. This early‐stage exploratory study strongly supports THBS1 as a candidate biomarker for chronic inflammatory enteropathy in cats.

**Abbreviations**
CE: chronic enteropathies
ECM: extracellular matrix
FCEAI: feline chronic enteropathy activity index
FDR: false discovery rate
fPLI: feline pancreatic lipase immunoreactivity
fTLI: feline trypsin‐like immunoreactivity
IBD: inflammatory bowel disease
LGAL: low‐grade alimentary lymphoma
MMP: matrix metalloproteinase
MS: mass spectrometry
PARR: polymerase chain reaction to assess antigen receptor rearrangements
T4: thyroxine
THBS1: thrombospondin‐1

## 1 INTRODUCTION

Chronic enteropathy in cats is 1 of the most common disorders of cats and is characterized by persistent or recurrent signs of gastrointestinal disease such as weight loss, vomiting, inappetence, and diarrhea.^1^, ^2^, ^3^ Food responsive enteropathy, idiopathic inflammatory bowel disease (IBD) and low‐grade alimentary lymphoma (LGAL) are forms of chronic enteropathies (CE) in cats with differing aetiologies.^1^, ^3^, ^4^ Intestinal biopsies obtained via gastrointestinal endoscopy or surgery for histopathologic assessment are the current standard required for definitive diagnosis.^1^, ^4^ When diagnoses are not reached with histopathological evaluation, additional diagnostic tests such as immunohistochemistry and clonality testing might be required in ambiguous cases.^1^ However, even with immunohistochemistry and clonality testing, definitive diagnoses are not always achieved.^1^, ^5^ There is a high rate of false positive samples with a specificity of 33% with clonality testing in cat samples.^5^, ^6^

Biomarkers have been investigated as potential diagnostic tests for CE with minimally invasive diagnostic methods.^7^ The most commonly used biomarkers in cats in clinical practice are serum cobalamin and folate which can be misleading for diagnosis of CE in cats if comorbidities are present.^1^ In humans, serum protein biomarkers are used to diagnose, monitor treatment response, and to differentiate between different forms of CE and therefore represent a novel, noninvasive, diagnostic, and monitoring tool for human IBD.^8^, ^9^, ^10^, ^11^, ^12^, ^13^ In cats, proteomics has been studied in several areas including chronic kidney disease, mammary carcinoma, pancreatic disease, osteoarthritis, and cat semen.^14^, ^15^, ^16^, ^17^, ^18^, ^19^ Given the extensive use of serum protein profiles in IBD in human medicine and studies in other areas of small animal medicine, serum proteomics might provide useful biomarkers in cats with CE.^8^, ^14^ The discovery of new proteomic biomarkers is a multi‐phase process which involves screening for potential biomarkers using an untargeted approach with a small sample size at the discovery phase followed by verification of selected proteins using targeted approaches with a larger sample size at the validation phase.^20^, ^21^ A recent study investigated the intestinal mucosal proteome in cats with 9 proteins found to be differentially expressed between healthy cats and cats with IBD and LGAL.^22^ However, western blot analysis did not confirm significant differential protein expression.^22^ The utility of serum proteomics in diagnosis of CE in cats has not been explored.

The aim of this exploratory study was to investigate changes in the serum proteomic profiles of cats with CE. This study provides useful preliminary data on the serum protein markers differentiating healthy cats from cats with CE. The ability to use a minimally invasive diagnostic method to assist in diagnosis of CE might reduce the need for more invasive diagnostics and anesthesia for pets with comorbidities and lead to reduced costs for pet owners.

## 2 MATERIALS AND METHODS

### 2.1 Animals and sample collection

Cases were recruited from 3 veterinary hospitals, including referral and primary care practices, between May 2019 and November 2020. Cats presented for evaluation of CE with clinical signs (vomiting, diarrhea, weight loss, or a combination of these signs) of at least 3 weeks' duration, and that were eventually diagnosed with CE/LGAL, were prospectively recruited for this study. Cat information including signalment, diagnostic tests, diet, previous and current treatment, and comorbidities was recorded. Cats with comorbidities such as pancreatitis, cholangiohepatopathy, urinary tract disease, and endocrinopathies, and cats without intestinal biopsies were excluded from further assessment. Diagnoses of CE were made on the basis of clinical signs, histopathology and exclusion of other causes of gastrointestinal manifestations, including metabolic disease, infection, parasitic disease, hepatic, and renal disease. The following diagnostic tests were performed: a complete blood count, a serum biochemistry profile, serum total T4 concentration, serum concentrations of cobalamin, abdominal ultrasonography and fecal flotation and PCR (feline coronavirus, *Tritrichomonas foetus*, *Cryptosporidium species*, panleukopenia virus, *Clostriudium perfringens*, *Giardia species*, *Salmonella species*, *Toxoplasma gondii*, *Campylobacter jejuni*, *Campylobacter coli*). Serum feline pancreatic lipase immunoreactivity (fPLI) and feline trypsin like immunoreactivity (fTLI) were measured in some cats. All the cats diagnosed with CE using endoscopic biopsies were scored using the feline chronic enteropathy activity index (FCEAI) which was calculated based on their clinical signs, clinicopathological, and endoscopic findings.^3^ Cats diagnosed via surgical biopsy were not FCEAI scored. All endoscopies were performed by a single board‐certified veterinary internist (LB). Eight out of 10 abdominal ultrasounds were performed by board‐certified veterinary radiologists from a single referral hospital while 2 ultrasound examinations were performed by general practitioners (cases recruited from primary care practices). All histopathological examinations of biopsied tissue samples were performed by board‐certified anatomic pathologists from different institutions and 7 cases were retrospectively reviewed following the WSAVA standards (MK). Controls were healthy cats that did not show clinical signs of gastrointestinal disease or weight loss confirmed with history collection and unremarkable physical examination findings. Some control cats were staff cats while others were enrolled during annual wellness health check at primary care practices. Blood samples were collected via jugular venipuncture, followed by centrifugation for serum separation. The serum was then collected into a serum tube and stored at −20°C before analysis.

### 2.2 Protein sample preparation

All serum samples were assessed for total protein concentration using the 2D Quant kit (Cytiva, Massachusetts, USA) as per manufacturer's instruction. Samples of 100 μg total protein were mixed in 50 μL AMBIC buffer (50 mM Ammoniumbicarbonate, 10 mM DTT, 2 M urea at pH 8) and trypsin digested at 25°C for 16 hours in a 1:100 enzyme‐to‐protein ratio based on the calculated serum protein concentration.^11^ Digestion was halted by acidification. Each sample was then dried to remove the AMBIC, reconstituted in 50 μL 0.1% formic acid and desalted and non‐peptide contaminants removed using C18 stage tips (Thermo Scientific, Illinois, USA) according to the manufacturer's recommendations except that the elution buffer consisted of 80% CH_3_CN, 0.1% Formic acid.

### 2.3 Mass spectrometry

Digested peptides were reconstituted in 10 μL 0.1% formic acid and separated by nano‐LC using an Ultimate 3000 HPLC and autosampler (Dionex, Amsterdam, Netherlands) and followed methods described previously.^23^ Briefly, the sample, 1.7 μL from 10 μL, was loaded onto a micro C18 pre‐column (300 μm × 5 mm, Dionex) with H_2_O:CH_3_CN (98:2, 0.1% TFA) at 10 μL min^−1^. After washing, the pre‐column was switched (Valco 10 port valve, Dionex) into line with a virgin fritless nanocolumn (75 μm i.d × 20 cm) containing reverse phase C18 media (1.9 μm, 120 Å, Dr. Maisch HPLC GmbH). Peptides were eluted using a linear gradient of H_2_O:CH_3_CN (98:2, 0.1% formic acid) to H_2_O:CH_3_CN (64:36, 0.1% formic acid) at 250 nL/min over 120 min. The QExactive (Thermo Electron, Bremen, Germany) mass spectrometer was run in DDA mode where a high voltage of 2000 V was applied to a low volume union and the column (45°C) positioned 0.5 cm from the heated capillary (275°C). A survey scan 350‐1750 m/z was acquired in the Orbitrap (resolution 70 000 at 200 m/z) with an accumulation target of 10^6^ ions, lock mass enabled and up to the 10 most abundant ions (AGC target set to 10^5^, minimum AGC target set to 1.5 × 10^4^) with charge states ≥+2 and ≤+6 sequentially isolated and fragmented. Samples were run as a single batch in a random order separated by a buffer injection H_2_O:CH_3_CN (98:2, 0.1% formic acid).

### 2.4 Protein characterization

Protein dataset‐peak lists were generated from raw files using Mascot Daemon v2.5.1 (Matrix Science, London, UK, www.matrixscience.com). All MS/MS spectra were searched against the Uniprot database (downloaded Jan 2021; 563 972 sequences) for protein identification with the following criteria: allowed 1 missed cleavage; variable modifications oxidation (M), deamidation (R), carbamidomethyl (C), and phosphorylation (S,T,Y); peptide tolerance, ±5 ppm; fragment tolerance, ±0.5 Da; peptide charge +2 to 4+; and enzyme specificity set to semi‐tryptic. A decoy database search was also performed. Scaffold Software (version 4.6.1, Proteome Software Inc., Portland, Oregon, USA) was used to record the protein profiles using spectral counting. Identifications were accepted if they could establish less than 5% false discovery rate (FDR) and contained at least 2 identified peptides per protein.^24^

### 2.5 Statistical analysis

Descriptive statistics of cat information including age, sex, weight, FCEAI and cobalamin concentrations were analyzed using R software. Continuous data with non‐normal distribution including age, weight and protein concentrations was compared and analyzed using the Mann‐Whitney *U* test. Sex status was analyzed using Chi‐square test. Data were Log 2 transformed using NCSS (version 9) software and normal distribution confirmed by D'Agostino's K‐squared test.

Changes in expression of protein (defined as fold change) were analyzed using Fisher's exact test with Benjamini‐Hochberg adjusted *P* values. Differences in fold change were considered statistically significant at *P* < .02, with a minimum fold change of ≥5.^25^, ^26^, ^27^ Fold change ratios and significance were calculated for CE/Control.

For bioinformatics analysis, the Gene Ontology concept was used to investigate molecular function, biological processes, and cellular components. The identified biological module groups were evaluated by their enrichment score and the significance of the module's enrichment was determined by AmiGo Panther analysis.^28^

Data were also analyzed with STRING version 11.5 database (https://string-db.org).^29^ Proteins identified with significant (and fold change >2) changes in expression were uploaded into STRING to map corresponding protein‐protein interactions. Graphical networks for these proteins were constructed based on their connectivity algorithms.

## 3 RESULTS

### 3.1 Cats

Fifty‐five cats were initially included: 29 cats with chronic signs of gastrointestinal disease and 26 control cats. After exclusion of cats with comorbidities, and cats without intestinal biopsies, a total of 31 cats were included in our proteomic study. Based on clinical signs, physical examination findings, exclusion of other gastrointestinal diseases, and available histopathology results, 10 cases were classified as CE. Four cats had endoscopic biopsies collected by gastroduodenoscopy and colonoscopy and 6 had surgical biopsies. Endoscopic biopsies were taken from the stomach, duodenum, ileum, and colon while surgical biopsies include stomach, duodenum, jejunum, ileum, mesenteric lymph node liver, or a combination of these tissues. The median number of biopsy specimens examined per site was 4 (range, 1‐11). Six cases were classified as lymphoplasmacytic enteritis (4 moderate, 2 moderate to severe), 3 cases had lymphoplasmacytic and eosinophilic enteritis (1 mild to moderate, 1 moderate, 1 moderate to severe) and 1 case had moderate to severe mixed inflammation. Nineteen cats classified as healthy controls were enrolled in this study. All cases had physical examination and history evaluated and were free of clinical signs of CE. Gastrointestinal histology was not available in these cats because of ethical concerns. Characteristics of cats are summarized in Table 1. All cats with CE had complete blood count and serum biochemistry profile, total T4 concentration and abdominal ultrasound performed. Fecal analyses were performed in 5 cats. fPLI was available in 4 cats and fTLI was available in 1 cat. Among the 10 cats with CE, 5 cats were receiving prednisolone treatment. One cat was concurrently treated with chlorambucil. All but 1 CE cat had been on dietary trials with hydrolyzed, novel protein or antigen‐restricted diets previously. Diets recorded at the time of sample collection included prescription hydrolyzed diets, novel protein diets or commercial cat foods. Six cats had immunosuppressant responsive enteropathy while only 1 cat was food responsive. For the remaining 3 cats, 1 cat was lost follow up, 1 cat died of septic peritonitis after surgical biopsy and 1 cat was euthanized because of gastrointestinal foreign body obstruction while on a dietary trial. This cat had recurrent foreign body obstruction due to pica which was likely related to CE. The dietary trial was performed after surgical biopsies collected during foreign body removal surgery. The cobalamin concentrations (n = 9) ranged from <150 to 1647 pmol/L (<203‐2232 ng/L) and the median was 717 pmol/L (972 ng/L). The 1 remaining cat had received cobalamin supplementation before referral, cobalamin concentration was therefore not measured. The median protein concentrations of samples in CE and controls measured using the 2D Quant kit were 85.3 mg/mL (range, 55.1‐140 mg/mL) and 101 mg/mL (range, 65.4‐144 mg/mL), respectively. There were no significant differences in age (*P* = .38), sex status (*P* = .64), and body weight (*P* = .61) between the CE and clinically healthy cats. There was also no significant different in protein concentrations (*P* = .10) between the CE and control cats.

**TABLE 1:** Characteristics of CE and healthy control cats by sex, age, weight, body condition score, breed, FCEAI, and cobalamin concentrations.

	CE (n = 10)	Controls (n = 19)
Age (years), median (range)	9.5 (2‐17)	9 (4‐16)
Weight (kg), median (range)	4.74 (2.67‐6.53)	4.80 (3.80‐6.50)
Body condition score, median (range)	4.5 (3‐6)	5 (5‐7)
Sex	5 males, 5 females	11 males, 8 females
Breed	3 DSH, 2 DMH, 2 Devon Rex, 1 Burmese, 1 Birman, 1 Norwegian Forest	14 DSH, 2 DMH, 2 sphynx, 1 Birman
FCEAI, median (range)	4.5 (4–6)^a^	‐
Cobalamin (ng/L) Median (range)	972 (<203‐2232)^b^ (RI: 339‐1626)	‐

Abbreviation: RI, reference interval.

^a^ Only the 4 cats with endoscopic biopsies had FCEAI score, other 6 cats had surgical biopsy.

^b^ Cobalamin only available in 9 cats.

Two additional cats were recruited and were classified as LGAL based on histopathology. One cat with LGAL had endoscopic biopsies via gastroduodenoscopy and colonoscopy and the other cat had surgical biopsies. Immunohistochemistry and PARR (polymerase chain reaction to assess antigen receptor rearrangements) were not performed. Both cats with LGAL were receiving prednisolone, and 1 was also receiving cyclophosphamide treatment.

### 3.2 Mass spectrometry

**FIGURE 1:**
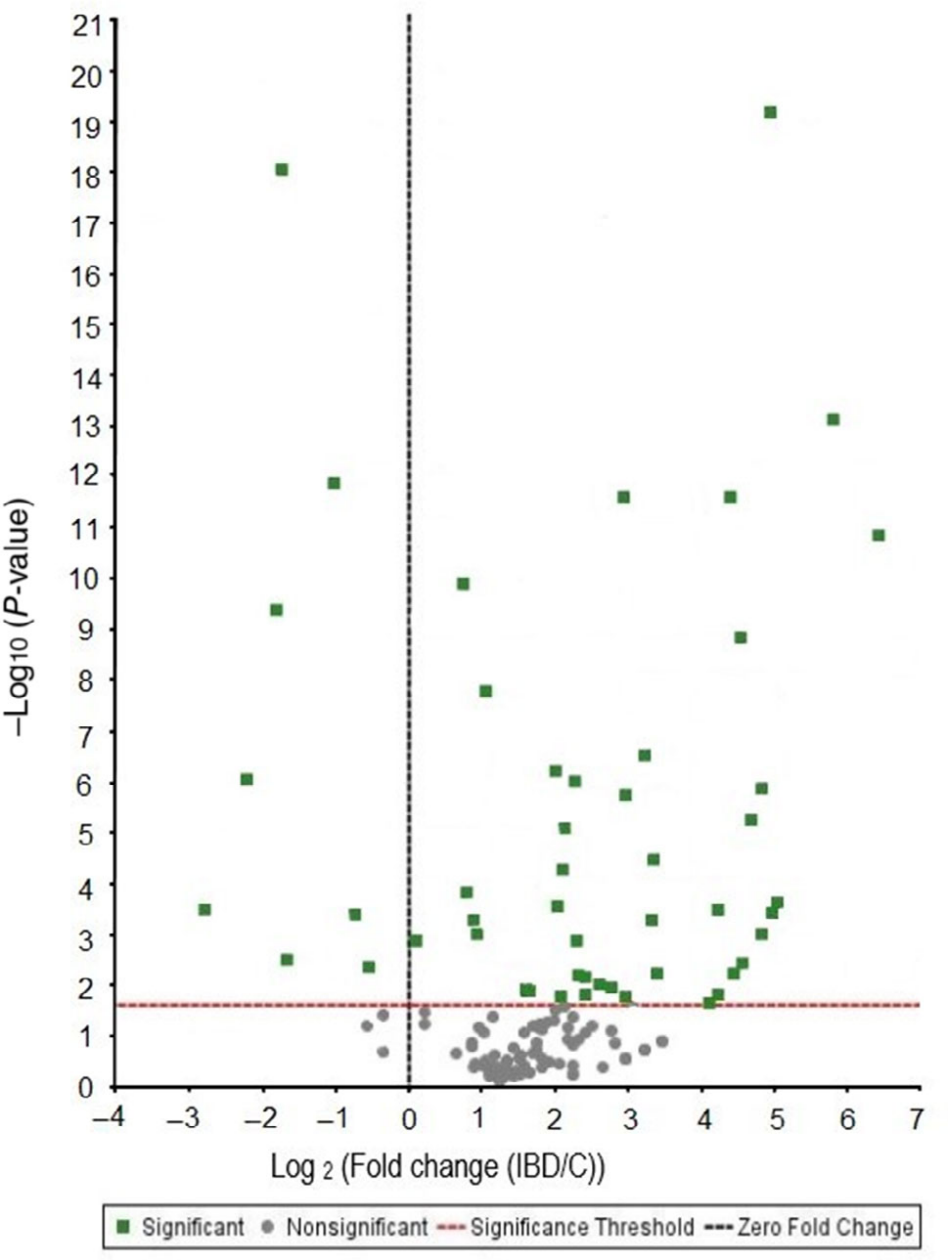
Volcano plot showing abundance graph of log 2‐fold change CE in reference to controls (CE/C) with statistical significance (Fisher's exact test) set to *P* < .02. X axis represents upregulated (>0) and downregulated protein (<0) abundance of cats with CE in reference to controls.

An average of 219 proteins were identified by mass spectrometry across multiple cats and phenotypes, of which 205 proteins were collectively found in both cats with CE and controls. Nine proteins were identified above MS thresholds in cats with CE only, while 5 were expressed in control cats only. Twenty‐six proteins were differentially abundant among the cats with CE and controls with fold change ≥5 (*P* < .02) (Table 2). Among these significantly modified proteins, 25 were upregulated while 1 protein was downregulated. Using log2 transformed fold change, the significantly changing proteins between control and cats with CE based on total spectral counts, 2 peptide identification and FDR of 5%, *P* ≤ .05 is shown in Figure 1. Proteins indicated by dots above the significance threshold and in particular highlighted proteins (green squares) were further explored.

**TABLE 2:** Consolidated and significant differential proteins between cats with CE and controls (CE/C) were found in 26 proteins with fold change ≥5 (*P* < .02).

Identified proteins	Accession number	Alternate ID	Fisher's exact test (*P* < .02)	Fold change (CE/C)	Log2 (fold change CE/C)
Coagulation factor V	Q9GLP1	F5	<.001	103	6.68
Thrombospondin‐1	P07996	THBS1	<.001	61.8	5.95
Heparin cofactor 2	P47776	SERPIND1	<.001	39.9	5.32
Apolipoprotein E	P0DSE0	APOE	<.001	39.0	5.28
GDH/6PGL endoplasmic bifunctional protein	P56201	H6PD	<.001	36.1	5.17
Antithrombin‐III	P41361	SERPINC1	<.001	33.3	5.06
Phosphatidylcholine‐sterol acyltransferase	Q08758	LCAT	<.001	32.3	5.01
Carboxypeptidase N catalytic chain	Q2KJ83	CPN1	<.001	31.4	4.97
Plastin‐2	P13796	LCP1	.003	28.5	4.83
Apolipoprotein C‐III	P0DSP0	APOC3	<.001	28.5	4.83
Inter‐alpha‐trypsin inhibitor heavy chain H3	P56652	ITIH3	<.001	27.6	4.78
Complement factor I	Q61129	Cfi	.01	26.6	4.73
Profilin‐1	P07737	PFN1	<.001	22.8	4.51
Apolipoprotein B‐100	P04114	APOB	.01	22.8	4.51
Coagulation factor IX	Q6SA95	F9	<.001	20.9	4.39
Tropomyosin alpha‐4 chain	P02561 (+2)	TPM4	.01	10.8	3.43
Insulin‐like growth factor‐binding protein complex acid labile subunit	P70389	Igfals	.01	9.03	3.17
Apolipoprotein C‐I	P0DM82	APOC1	<.001	8.21	3.04
Transthyretin	P12303	TTR	.01	7.92	2.99
Hemoglobin subunit beta	P41328	HBB	.01	6.49	2.70
Serum paraoxonase/arylesterase 1	P27169	PON1	.01	6.27	2.65
Complement	P01026	C3	.01	5.95	2.57
Fibronectin (Fragment)	Q28275	FN1	<.001	5.32	2.41
Actin, cytoplasmic 1	P84336	ACTB	.001	5.27	2.40
Hemopexin	Q5R543	HPX	.015	5.13	2.36
Beta‐enolase	P15429 (+1)	ENO3	<.001	−5.79	−2.53

### 3.3 Gene ontology analysis

**FIGURE 2:**
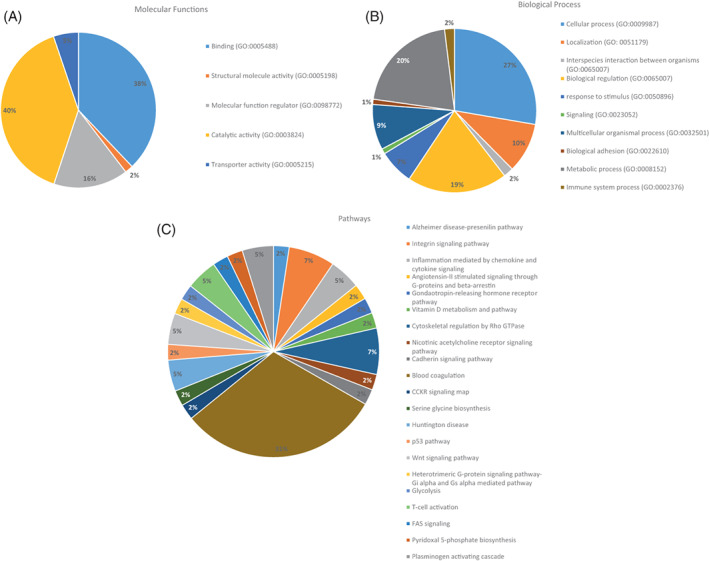
(A) Molecular functions of ≥|2| fold increased protein in abundance and significance in cats with chronic enteropathies (CE) compared to controls. (B) Biological process of ≥|2| fold increased protein in abundance and significance in cats with CE compared to controls. (C) Differences in pathways between cats with CE and controls by significance and ≥|2| fold change with gene analysis.

Gene ontologies were categorized according to enriched proteins of significance with ≥|2| fold change of CE:Controls as shown in Figure 2A‐C analysis results were divided into molecular function, biological process, and pathway. The results of molecular function, biological process and pathway for proteins with a significant difference in expression between CE and control cats (and ≥|2| fold change) are shown in Figure 2A‐C, respectively. Most proteins differentiating cats with CE from controls have catalytic activity, binding functions or are regulatory proteins (Figure 2A), involved in cellular, regulatory or metabolic processes (Figure 2B). Pathways already known to be involved in human IBD lesions include cytoskeletal regulation, integrin and cytokine signaling and plasminogen and T cell activation pathways (Figure 2C).

**FIGURE 3:**
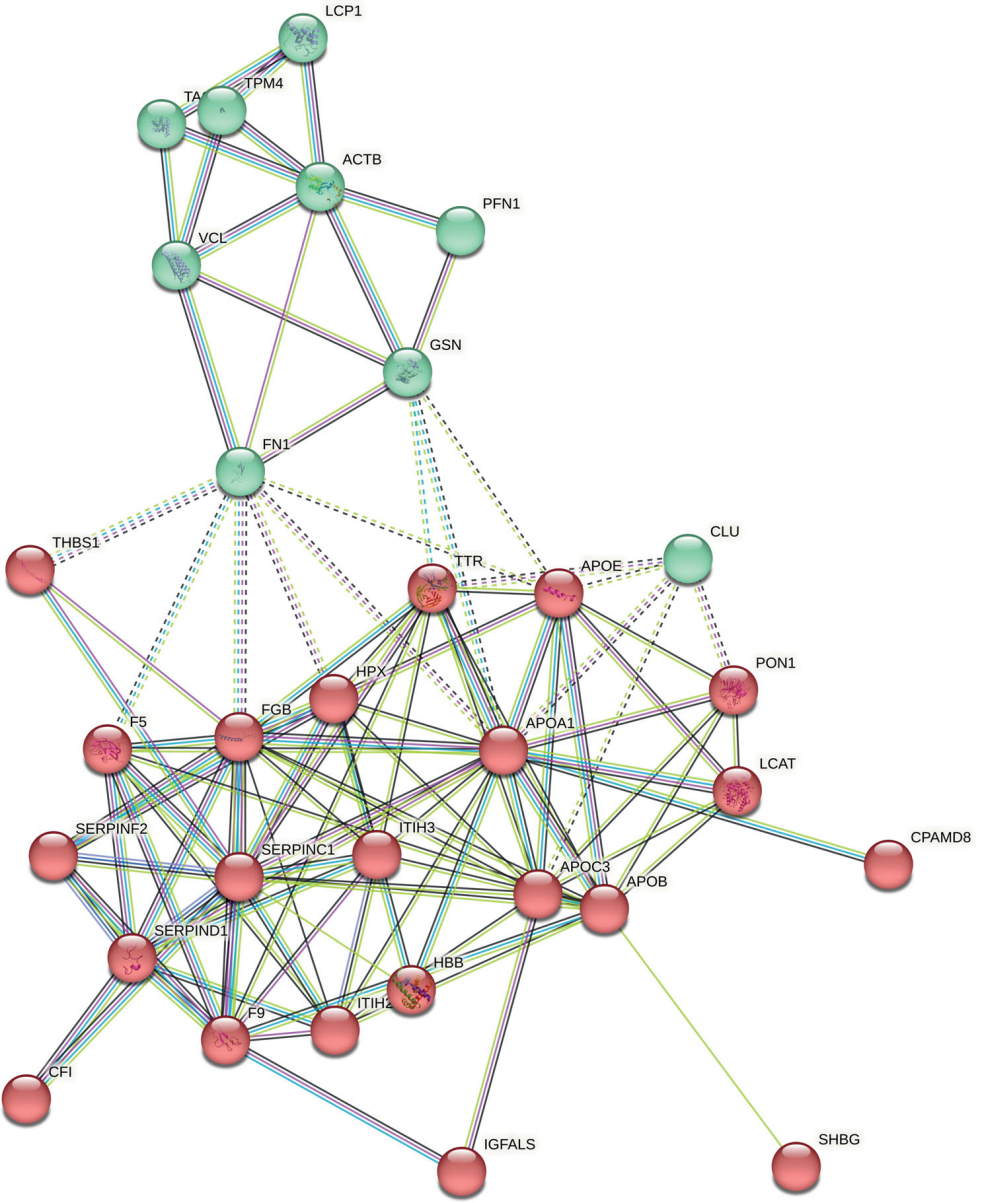
Protein‐protein interaction network for differentially expressed proteins between cats with chronic enteropathies and controls shows 2 distinct clusters (red and green) representing the involvement of the (a) extracellular matrix (ECM) and (b) altered coagulation, lipid regulation in the inflammatory state. ACTB, actin; APOA1, apolipoprotein A1; APOB, apolipoprotein B; APOC3, apolipoprotein C3; APOE, apolipoprotein E; CLU, clusterin; CPAMD8, C3 and PZP‐like alpha‐2‐macroglobulin domain containing protein 8; F5, coagulation factor V; F9, coagulation factor IX; FGB, fibrinogen beta chain; FN1, fibronectin; GSN, gelsolin; HBB, hemoglobin subunit beta; HPX, hemopexin; ITIH2, inter alpha trypsin inhibitor heavy chain H2; ITIH3, inter alpha trypsin inhibitor heavy chain H3; LCAT, phosphatidylcholine‐sterol acyltransferase; LCP1, plastin 2; PFN1, profilin 1; PON1, serum paraoxonase/arylesterase 1; SERPINC1, antithrombin‐III; SERPIND1, heparin cofactor 2; SERPINF2, alpha‐2‐antiplasmin; TAGLN2, transgelin‐2; THBS1, thrombospondin 1; TPM4, tropomyosin alpha‐4 chain; TTR, transthyretin; VCL, vinculin.

Using the STRING algorithm, the interactions between proteins including physical and functional associations were analyzed (Figure 3). K‐means was used to identify 2 prominent clusters of proteins significantly changing between cats with CE and controls with ≥|2| fold change. The 2 clusters demonstrate the involvement of the extracellular matrix (inclusive of THBS1, profilin, transgelin, fibronectin, vinculin, and actin) and altered lipid metabolism in an inflammatory state (inclusive of the apolipoproteins, serpins, serum paraoxonase/arylesterase 1 [PON1] and inter‐alpha‐trypsin inhibitors).

**FIGURE 4:**
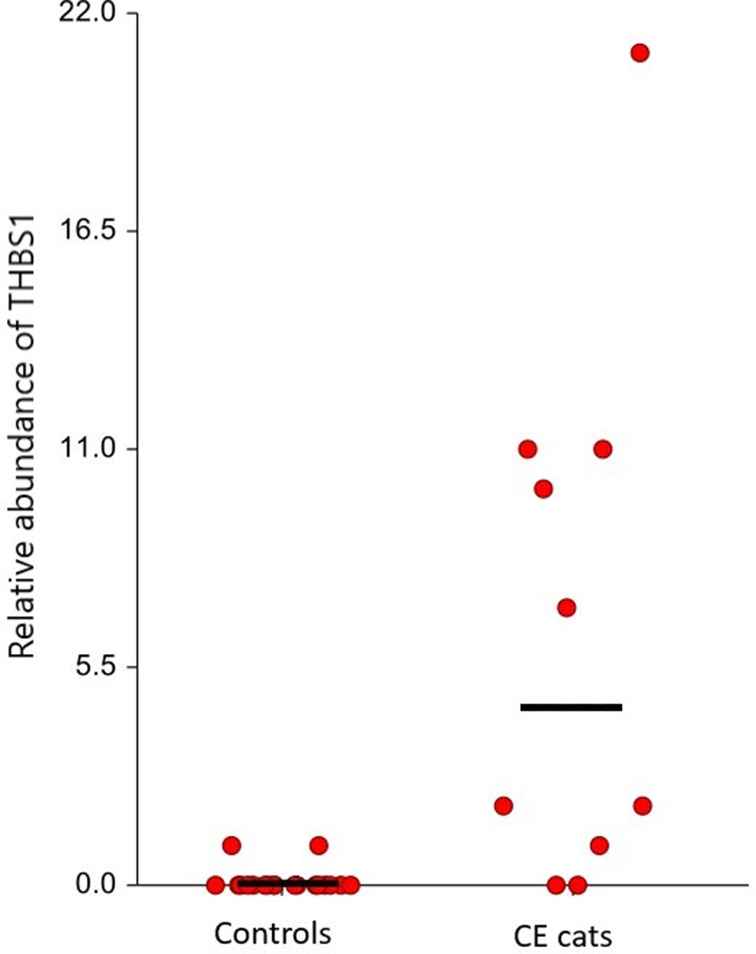
A dot plot showing the relative abundance based on spectral count of THBS1 in individual control and cats with chronic enteropathies (CE). The median is illustrated by the black line, median for cats with CE is 4.5 (range, 0‐21) and median for controls is 0 (range, 0‐1).

Further evaluation of THBS1 abundances in individual cats better shows the significant increase in THBS1 in cats with CE (median 4.5, range 0‐21) compared to controls (median 0, range 0‐1) (*P* < .001), and notably lower abundance in 2 cats with LGAL (median 0.5, range 0‐1). Comparative statistics between cats with CE and LGAL were not performed due to the small sample size of cats with LGAL. A dot plot (Figure 4) showed the relative abundance based on spectral count of thrombospondin in individual control and cats with CE. Linear regression of controls and cats with CE gave a Pearson correlation of 0.597 with *P* < .001. Within the group of cats with CE, 5 cats were receiving prednisolone treatment. There was no significant correlation in our study related to prednisolone treatment of cats with CE (Pearsons correlation was 0.232).

## 4 DISCUSSION

Proteomic analysis allows large scale detection and rapid identification of proteins of pathological significance.^30^ Our results show significant differences between the serum protein profiles of cats with CE and healthy cats. Twenty‐six proteins were considered statistically significantly different (*P* < .02 and greater than |5| fold abundance change) in confirmed cats with CE compared with healthy controls. Differentially abundant proteins included several proteins involved in cellular processes, proteins with binding‐activities, modifying enzymes, and metabolic enzymes. Although further studies are required for validation, these preliminary data indicate the potential for using serum to identify disease related proteins from cats with CE. Proteins with the greatest abundance discrepancies between controls and cats with CE included coagulation factors, thrombospondin‐1, serpins, antithrombins, apolipoproteins, complement, hemopexin, and fibronectin. These proteins are involved in remodeling and regulation of the extracellular matrix, inflammation, and lipid metabolism; a common finding with human IBD.^31^, ^32^ Many of these proteins have important roles in the maintenance of the intestinal environment, for which control and regulation of mucosal tolerance is critical.^31^

Among the 26 proteins reported here, thrombospondin‐1 (THBS1) is of particular interest in the pathology of CE. Thrombospondins are a family of secreted glycoproteins that are important in maintaining the extracellular matrix (ECM) structure and regulation of cellular phenotype.^33^, ^34^ It has important functions in inflammation, clotting, anti‐angiogenesis, proliferation, cell attachment, and motility.^33^, ^34^ THBS1 level increases have been noted in humans with IBD and has been extensively studied as a protein biomarker in diagnosis and treatment monitoring.^33^, ^35^, ^36^ In addition, its anti‐angiogenic property has been investigated as a potential new therapeutic approach to intestinal inflammation in IBD in people and cancer in dogs.^37^, ^38^, ^39^ In other species, THBS1 was confirmed by ELISA and Western blot to be differentially expressed in serum samples of mice with experimentally induced mid‐ to late‐stage IBD compared to those with early non‐inflamed stage IBD.^40^ THBS1 has not previously been explored in cats. Lower abundance of THBS1 was observed in 2 cats with LGAL when compared to cats with CE, with the limited number of cats these findings are obviously highly preliminary.

The data reported here show potential for THBS1 to be used as a diagnostic test in enteropathies in cats, where the diagnosis typically relies on invasive surgical or endoscopic intestinal biopsies. However, further investigation of this preliminary finding using western blot analysis, parallel reaction monitoring technique and data analytical validation are required to test the feasibility of using THBS1 in diagnosis of CE in cats. Additional cohort studies between cats with LGAL and CE, food responsive and immunosuppressant responsive enteropathies are warranted. THBS1 is a glucocorticoid responsive protein in humans and was elevated in human patients being treated with prednisolone and with Cushing's syndrome.^41^, ^42^ In our study, no correlation was found between THBS1 abundance and cats treated with prednisolone in all groups.

A further finding in the study reported here was the involvement of coagulation factors in enteropathies. Coagulation factor V showed a significant difference with >100‐fold increase in relative abundance (*P* < .001) in cats with CE compared to controls in our analysis. Coagulation factor V is a protein of the coagulation system and acts as a non‐enzymatic cofactor for activated factor X to form the prothrombinase complex which contributes to generation of thrombin.^43^ The relationship between hemostasis and CE is complex. Increased risk of thromboembolism and platelet activation have been observed in IBD in humans.^44^ Further, increased generation of thrombin has been demonstrated in human IBD patients compared to controls and was thought to be associated with a partial loss of function of the natural anticoagulant pathways.^44^, ^45^ The imbalance between procoagulant and anticoagulant pathways with the changes in the fibrinolytic system are thought to contribute to thromboembolism in human IBD.^44^ A hypercoagulable state has also been detected by thromboelastography in dogs with normoalbuminemic and hypoalbuminemic chronic inflammatory enteropathy.^46^, ^47^ Although the mechanism is not completely understood, suggested causes of thrombosis in protein losing enteropathy in dogs include systemic inflammation, loss of antithrombin III, platelet hyperaggregation, hyperfibrinogenaemia, vascular compromise and altered vitamin K absorption.^48^ Antithrombin has been shown to be a positive acute phase protein in cats and was increased during inflammation.^49^ In our study, coagulation factor V, IX, antithrombin III and heparin cofactor 2 were upregulated in cats with CE compared to control cats. This finding may be related to hypercoagulable state in chronic inflammatory enteropathy. Given that hypercoagulable state can be seen in other diseases,^50^ despite having the highest fold change in abundance, coagulation factor V was not selected for further analysis as a candidate biomarker. Since no studies have investigated hypercoagulability in CE in cats, further studies are needed to determine the association of hemostasis and CE in cats.

The relationship between proteins in our study was mapped using the STRING algorithm (Figure 3), and the findings were dominated by the involvement of ECM proteins. ECM remodeling is a hallmark of IBD in humans.^51^ Recent studies support the role of the ECM as an active component in promoting inflammation in the pathogenesis of IBD.^51^ The alteration of ECM in IBD is characterized by inflammatory mediators and activated matrix metalloproteinase‐9 (MMP9), which increases intestinal permeability, epithelial apoptosis and loss of goblet cells in colitis in humans.^51^ In dogs, upregulation of mucosal active MMP2 and MMP9 was found in the intestines of dogs with CE compared to healthy dogs.^52^ In addition, intestinal stricture formation and fibrosis have been linked to increased production in specific components of the ECM including fibronectin and collagens in IBD.^51^ In our study, many ECM components such as fibronectin, inter‐alpha‐trypsin inhibitor heavy chains, profilin‐1 and actin were upregulated in cats with CE compared to healthy cats. Further, as shown in the data reported here (Figure 3), the interrelationship between inflammation, ECM and platelets contributes to the upregulation of protein clusters involving ECM and altered coagulation. The dysregulation of vascular ECM in human IBD patients has been found to promote adhesion and extravasation of leukocytes and platelets in the endothelium.^51^ The cell adhesion molecules such as E‐selectin interact with components of the ECM (fibronectin, collagens, laminin) to regulate the recruitment of circulating leukocytes and control endothelial permeability.^51^

An apparent change in lipid metabolism, with upregulation of apolipoproteins A‐I, B‐100, C‐I, C‐III, and E, was identified in cats with CE compared to control cats in our study. Of these proteins apolipoproteins B‐100, C‐I, C‐III, and E showed fold change ≥5. In cats, the distribution of serum lipoproteins and apolipoproteins is unlike that observed in humans. Lipoproteins in the cats are larger and richer in triglycerides compared to humans.^53^ Disturbances in lipid protein, lipoprotein concentrations and composition are observed in people with Crohn's disease^54^ Changes in cholesterol and lipoprotein metabolism in IBD include hypocholesterolaemia, hypertriglyceridemia and decreased high density lipoprotein levels.^32^ In a study investigating serum metabolomic profiles in human IBD patients, lipid metabolism, including bile acid pathways, was found to be significantly altered in human IBD patients compared to healthy controls.^55^ The change in bile acid metabolism has been well described in IBD in both humans and dogs and is associated with malabsorption and dysbiosis in IBD dogs, with decreased conversion of primary bile acids to secondary bile acids from the lack of *Clostridium hiranonis*.^56^, ^57^ Another reason for the alterations in lipid metabolism might be that the active inflammatory state in IBD leads to elevated energy expenditure with enhanced protein catabolism and lipid utilization.^55^

In the study reported here, cats with CE had increased levels of other known acute phase reactants such as complement proteins C1 and C3. The complement system comprises of many plasma proteins that function as receptors or regulators of complement activation through 3 activation pathways: the classical pathway, lectin pathway, and the alternative pathway.^58^ Complement activation leads to the formation of C3 convertase which is the major and most abundant component in the complement cascade.^58^ Hyperactivation of complement has been reported in chronic inflammatory diseases and immunological diseases in people.^58^, ^59^ Furthermore, in people with Crohn's disease, alterations in complement cascade and innate immune response have been described.^60^ Serum C3 and C4 complement components were increased in human patients with Crohn's disease, with higher levels found in human patients with active disease compared to inactive disease.^61^ The increased fold change in complement proteins in cats with CE in our study could be related to the immunological basis of IBD or inflammation. Nevertheless, further studies are needed to establish the link between the complement system and CE in cats.

There are several limitations in our study. First, the small sample size was a limiting factor. Although differences in protein profile were observed between cats with CE and LGAL, we did not perform further analysis comparing the 2 groups due to the small sample size of cats with LGAL and therefore a definitive conclusion of difference in protein profiles between CE and LGAL cannot be drawn. Second, as the management of each case was at the discretion of the attending veterinarian, not all diagnostic tests were performed in each cat and treatment was not standardized. Specifically, fecal PCR, flotation, fPLI and fTLI results were only available for some cats. Although our analysis did not show any correlation in THBS1 abundance in cats treated with prednisolone, we cannot completely exclude an effect of glucocorticoids or other medications on our proteomic result. Last, although the use of proteomics allows rapid identification in a broad spectrum of proteins simultaneously, the use of serum proteomics was untargeted and identified large numbers of proteins which can be difficult to overcome in a standard shot‐gun proteomics experiment without intensive fractionation of the samples. Nevertheless, only the proteins with the most significant fold changes were included in our analysis. Overall, the data reported here indicating increased THBS1 abundance in cats with CE was strong; further verification of THBS1 abundance using targeted techniques like parallel reaction monitoring and western blot analysis are indicated in cats with CE for repeat.

Proteomic approaches with minimal fractionation have identified potentially useful markers of chronic inflammatory enteropathy in cats. The discovery and assessment of new protein candidates, particularly including THBS1 in cats with CE, could lead to novel testing modalities to diagnose and monitor treatment response in CE. Given the exploratory nature of this proteomic study, further studies with larger sample sizes and the verification of proteomic biomarkers using targeted techniques will be required before proposing this modality for clinical use.

## CONFLICT OF INTEREST DECLARATION

Authors declare no conflict of interest.

## OFF‐LABEL ANTIMICROBIAL DECLARATION

Authors declare no off‐label use of antimicrobials.

## INSTITUTIONAL ANIMAL CARE AND USE COMMITTEE (IACUC) OR OTHER APPROVAL DECLARATION

Approved by the Animal Ethics Committee at the University of Sydney (project number 2019/1548).

## HUMAN ETHICS APPROVAL DECLARATION

Authors declare human ethics approval was not needed for this study.
